# Survey-derived best management practices for backyard beekeepers improve colony health and reduce mortality

**DOI:** 10.1371/journal.pone.0245490

**Published:** 2021-01-15

**Authors:** Kelly Kulhanek, Nathalie Steinhauer, James Wilkes, Michaela Wilson, Marla Spivak, Ramesh R. Sagili, David R. Tarpy, Erin McDermott, Andrew Garavito, Karen Rennich, Dennis vanEngelsdorp

**Affiliations:** 1 Department of Entomology, University of Maryland, College Park, Maryland, United States of America; 2 Department of Computer Science, Appalachian State University, Boone, North Carolina, United States of America; 3 Department of Entomology and Plant Pathology, University of Tennessee, Knoxville, Tennessee, United States of America; 4 Department of Entomology, University of Minnesota, St. Paul, Minnesota, United States of America; 5 Department of Horticulture, Oregon State University, Corvallis, Oregon, United States of America; 6 Department of Entomology & Plant Pathology, North Carolina State University, Raleigh, North Carolina, United States of America; University of Alberta, CANADA

## Abstract

Honey bee colony losses in the US have exceeded acceptable levels for at least a decade, leaving beekeepers in need of management practices to improve colony health and survival. Here, an empirical Best Management Practice (BMP) regimen was tested, comprised of the top four management practices associated with reduced colony mortality in backyard beekeeping operations according to Bee Informed Partnership Loss and Management survey results. Seven study locations were established across the US, and each location consisted of ten colonies treated according to empirical BMPs and ten according to average beekeeping practice. After 3 years, colonies treated according to empirical BMPs experienced reduced *Varroa* infestation, viral infection, and mortality compared to colonies managed with Average practices. In addition, BMP colonies produced more new colonies via splits. The colonies under Average practices were given chemical *Varroa* treatments only once per year, and thus spent more months above economic threshold of 3.0 mites/100 bees. Increased time spent above the economic threshold was significantly correlated to both increased viral infection and colony mortality. This study demonstrates the cumulative effects of management and colony health stressors over months and years, especially the dire importance of regular *Varroa* monitoring and management.

## Introduction

Honey bees are the most economically important pollinators in the world, providing billions of dollars in pollination services [1–3]. However, beekeepers consistently lose more colonies each year than they deem acceptable [4–9], and the need for pollination units has grown more rapidly than the supply of honey bee colonies [10]. Thus, beekeepers struggle to keep their operations viable and provide sufficient colonies for crop production.

Research has identified many factors contributing to the taxing rates of colony mortality [11]. The parasitic mite, *Varroa destructor*, causes direct damage via feeding wounds [12–14] and vectors a suite of viruses [15, 16]. Prolonged exposure to pesticides reduces a colony’s ability to combat other stressors [17, 18]. Poor nutrition further impacts colony health, particularly as landscapes are converted to monocultures that provide no or poor food resources [19]. While these factors may not kill colonies in isolation, in concert these stressors can interact to manifest colony death [11, 20]. Over the past decade, substantial research has focused on identifying these stressors and assessing their impacts. More recently, scientists have begun to investigate interactions between and among stressors to better understand colony experiences in real-world settings [21–23].

After identifying risk factors, the logical next step in an epidemiological challenge is to develop preventative strategies. Beekeepers have an opportunity to mitigate the effects of colony health stressors through the application of good beekeeping management practices. For example, beekeepers can provide colonies with supplemental food when natural pollen and nectar sources are scarce [7]. Additionally, interrupting *Varroa* population growth with various control measures is often required to reduce colony mortality [24]. For colonies and apiaries, it can be challenging to determine the effectiveness of these and other management practices because of multiple interacting health stressors [11]. Science-based management recommendations can help beekeepers avoid using trial and error to reduce colony mortality.

Multiple groups have conducted surveys on colony losses and beekeeping management around the world (Germany: [25]; Canada: [26, 27] Europe: [28, 29]). The Bee Informed Partnership (or BIP; beeinformed.org) has conducted an annual Loss and Management Survey of US beekeepers since 2010. The survey consists of over 80 questions about the number of colonies lost and management practices employed by a given operation over the previous year. Survey methods and results are published annually (reviewed in [7]). In total, the survey has collected over 50,000 responses, and it has built the largest database of colony loss and management information in the world. These data can be analyzed to assess the effectiveness of management practices as they relate to reduced colony mortality.

One practice consistently associated with reduced mortality is *Varroa* control. Beekeepers who control *Varroa* consistently lose fewer colonies annually [24, 30]. Despite clear evidence of their benefits, only 48% of backyard beekeepers (beekeepers with 1–50 colonies) have reported using *Varroa*-control measures over the duration of the BIP survey. While more backyard beekeepers report controlling for *Varroa* every year (up to 78% of backyard beekeepers in 2018), there are many “treatment-free” beekeepers who do not employ effective mite-control strategies [31, 32]. Further, backyard beekeepers who employ control measures typically only do so once per year [24], which is likely insufficient to reduce *Varroa* populations below economic thresholds. Backyard beekeepers experience the highest levels of colony loss each year [7], and improved *Varroa* control likely can reduce this mortality rate.

A full analysis of observational survey data was conducted to identify management practices that, if adopted, were predicted to have the largest reduction in colony loss rate. The top five of these empirical best management practices [BMPs; 33] were developed for four different beekeeper demographics (southern backyard, northern backyard, stationary professional, and migratory professional). Four of the top five empirical BMPs were the same for northern and southern backyard beekeepers. However, before recommending these four practices to beekeepers, they needed to be field-tested to assess their effects on colony health and mortality. To this end, a 3-year study was conducted to assess the effectiveness of these four BMPs. It was hypothesized that apiaries maintained according to the four empirical BMPs would reach larger colony sizes, exhibit better brood patterns, and experience fewer queen events. BMP apiaries were also hypothesized to experience lower *Varroa*, *Nosema*, and pathogen loads, reduced mortality, and produce more honey and colony splits.

## Methods

### Management practices

This experiment compared two different management regimes (Average vs. BMP; Table 1) with four categories of management practices: action on deadouts (beekeeping term for colonies that die), *Varroa* control frequency, method for starting new colonies, and comb-culling technique. The BMP regime was derived from a combination of expert recommendations and survey results in Steinhauer et al., 2020. Beekeeper’s survey responses were scored on how well they aligned with expert recommendations. Beekeepers with higher scores (more aligned with expert recommendations) experienced significantly reduced winter colony loss, indicating that the expert’s opinions were correct. Bootstrapped sensitivity analyses were performed to identify which management practices had the greatest effect on colony loss. The BMP regime in this study corresponds to the expert recommendation for the top four practices that most affected colony loss. The “Average practice” regime was derived from BIP Loss and Management Survey data as the most common practice employed by backyard beekeepers in the same four categories.

**Table 1:** Average practices vs. BMPs to be tested in the field.

	*Average Practice*	*BMP*
***Action on deadouts***	Store equipment for later use	Reuse equipment immediately by adding to living colonies or using for a split
***Varroa control frequency***	Apply miticides once in fall	Monitor monthly and apply miticides when above 3.0 mites/100 bees
***Starting new colonies***	Packages	Make splits when possible and buy nucs if splits impossible
***Comb culling technique***	Do not treat old brood comb before reuse	Freeze old brood comb before reuse

The only differences in management between Average and BMP groups were in the four categories of practices being tested, performed as follows. All other apiary management (*e*.*g*., feeding, requeening, honey harvest) was performed on an as-needed basis according to standard beekeeping practices and was kept consistent between the two groups.

Action on deadouts refers to how beekeepers respond to dead colonies discovered during the active season. The Average practice is to remove such equipment from the apiary and store it for later use, typically the following spring when a new colony is established. The empirical BMP associated with the lowest winter loss rate is to reuse that equipment immediately, either by making a new colony (split) using the equipment or by adding the boxes to another colony that needs more space. In reality, this BMP is difficult to enact because of the seasonality of discovering dead colonies (typically late fall), which does not correspond with the seasonality of needing equipment for new or expanding colonies (early summer). Additionally, caution should be exercised when introducing equipment from dead colonies to living ones, as it is possible to spread disease this way. Regardless, the practice of immediately reusing equipment was highly associated with lower winter mortality, making it one of the top 4 practices to be tested in this study. Deadout equipment was reused immediately when colonies in the same yard were available to receive additional boxes. If no colonies needed additional boxes, combs were frozen, stored, and frozen again before reuse the following spring.

*Varroa* control frequency refers to the frequency with which *Varroa* populations are managed. The Average practice is to apply miticides to the colony once per year in fall (typically in August or September). The BMP is to monitor *Varroa* on a monthly basis and employ control measures whenever a single colony in the apiary exceeds 3.0 mites/100 bees. This practice was followed strictly throughout the study for the BMP colonies at each location. The choice of specific miticide was left to the discretion of researchers in each state, as miticides have specific temperature and brood requirements, and honey contamination risks. Once a colony exceeded the threshold of 3.0 mites/100 bees, miticides were applied to all colonies within that apiary, in accordance with expert recommendations.

Starting new colonies refers to the manner by which a new colony is formed at the beginning of the beekeeping season. The average hobbyist beekeeper starts new colonies by purchasing packages. The empirical BMP is to start new colonies by making splits from successfully overwintered colonies. If insufficient colonies are available to split, then purchasing nucleus colonies is the next best option. In the spring of 2016, all colonies were started from packages installed on new plastic foundation to equalize the starting conditions of both management groups. After initial installation, if a colony died over the course of the year, it was not replaced until the following spring. In 2017 and 2018, new colonies installed in the spring came from packages in Average apiaries and splits in BMP apiaries. Apiaries were always replenished to a size of ten colonies each. If an insufficient number of BMP colonies survived the winter to make splits to reach ten colonies, local nucleus colonies were purchased.

Finally, comb culling refers to how brood comb is managed before it is reused in a new colony. Beekeepers often have a stock of old brood combs, typically from colonies that died previously or shrank in population, allowing a secondary empty brood box to be removed. These combs are later reused by the beekeeper, either by adding to a growing colony that needs an additional brood box or installing a new colony into it the following spring. Beekeepers sometimes treat this old comb to kill persistent *Nosema* spores, small hive beetle, or wax moth adults or larvae by using chemicals (*e*.*g*., paradichlorobenzene crystals or acetic acid), irradiation, or freezing. Most hobbyist beekeepers do not treat this brood comb before reusing it in a new colony. However, the empirical BMP is to freeze this comb at -20°C for a minimum of 24 hours prior to adding it to a new colony. In this study, all brood combs added to BMP apiaries were frozen prior to use, while combs used in Average apiaries were stored at ambient temperature.

This practice may seem at odds with the BMP of reusing deadout equipment immediately, and a beekeeper may wonder if it is better to reuse comb immediately or freeze the comb before reuse. As these practices were 2 of the top 4 practices that most affected winter mortality, they both had to be performed for this study. To this end, if a dead colony was discovered in a BMP apiary and the equipment could not be immediately reused, the combs were frozen immediately and then again before being added to a new colony. In other words, if the comb left the apiary, it was frozen before being re-introduced.

### Apiaries

**Fig 1:**
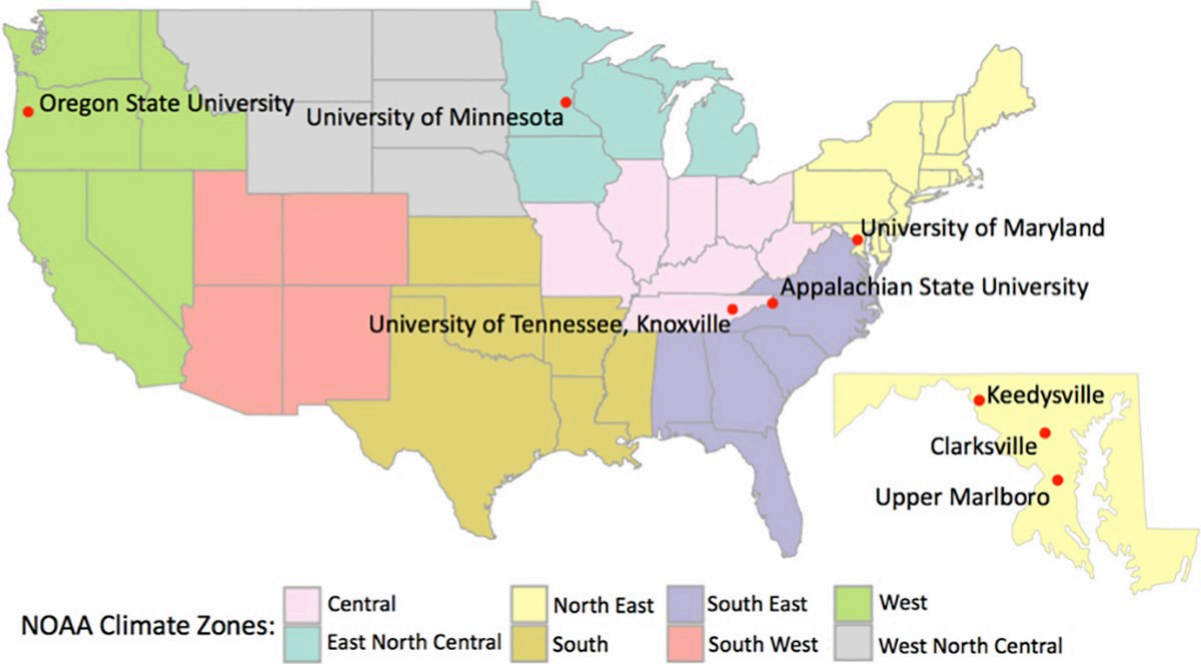
Map of apiary locations and corresponding NOAA climate zones.

This experiment was conducted at seven locations in five states across the US: Minnesota, Maryland, North Carolina, Oregon, and Tennessee (GPS coordinates of study sites can be found in S1 Table). Apiaries were maintained on university property or author’s personal property, so no special permits were necessary. Each state represented a different climatic region as designated by the National Oceanic and Atmospheric Administration (NOAA; [34] and was chosen to test the effectiveness of empirical BMPs in different climates (Fig 1). Each location divided 20 colonies into two groups of ten colonies each. One group was treated according to Average beekeeping practices, and the other was treated according to empirical BMPs defined above. The two groups were separated by 10–50 meters to minimize drift of bees between management groups at each location. Microclimates of the colony groups (*e*.*g*., hours of shade, direction of colony entrance) were kept as similar as possible. Apiaries were established in the spring of 2016 and maintained until the spring of 2019. Each colony was established from packages on new plastic foundation to minimize initial differences in colony strength.

### Sampling

All colonies in this study were monitored from spring 2016 through spring 2019. Each year, colonies were inspected and sampled once per month for 6 months from spring to fall. The actual months when colonies were sampled varied somewhat based on weather and climate in each region. For example, in 2016 Minnesota colonies were sampled from April to September, and North Carolina colonies were sampled from June to November. In all analyses, only data from May to October were used for simplicity of comparison between groups.

Each inspection included a colony strength assessment and record of the typical metrics of frames of bees, queen status, and brood pattern [35]. Frames of bees, a measurement of colony size, was evaluated according to standard methods [35]; one deep frame completely covered in adult bees on both sides was counted as one frame of bees. Mediums frames, if used, were counted as 2/3 of a full deep frame. Brood pattern was evaluated on a scale of 1–5, a 5 being a frame of continuously capped brood. Brood pattern is a standard colony health metric used by beekeepers, where better brood patterns are considered indicative of queen and brood health. Queen status was judged as one of six options: queen seen, queen-right (queen not seen but fresh eggs observed), virgin queen, drone layer, queen not seen (no queen or fresh eggs seen but seems otherwise queen right), or queen-less (clearly no queen present). If a colony experienced a queen issue, attempts were made to rectify it (*e*.*g*., adding a new queen or frame of eggs) but occasionally queen issues contributed to colony mortality.

A sample of adult bees was also taken from each colony at each sampling event. Approximately 300 adult bees were taken from a frame with partially capped brood and placed into a saltwater bottle. Super-saturated saltwater (1.13 kg salt per 3.79 liters water) was used in lieu of alcohol for ease of shipping, and all samples were processed before any decay occurred. Each participating researcher mailed their samples to the bee diagnostics lab at University of Maryland, where samples were processed for *Varroa* (mites/100 bees) by shaking and *Nosema* (millions of spores/bee) by microscope according to standard methods [36, 37].

A separate sample was taken from each colony for testing of viruses three times per year (spring, mid-summer, and fall). The precise timing of these samples varied based on regional climate, and only two samples were taken in the first year (mid-season and fall) as colonies were not established well enough to support an extra sample in spring. Viral sampling consisted of placing approximately 100 bees from a frame with partially capped brood into a 50 mL Eppendorf tube. The tubes were immediately placed on dry ice and kept at -80°C until they could be shipped on dry ice to the North Carolina State University Queen & Disease Clinic for processing. Samples were tested for copy numbers of the following viruses: Acute Bee Paralysis Virus (ABPV), Black Queen Cell Virus (BQCV), Chronic Bee Paralysis Virus (CBPV), Deformed Wing Virus A (DWVA), Deformed Wing Virus B (DWVB), Israeli Acute Paralysis Virus (IAPV), Lake Sinai Virus (LSV), *Trypanosoma* spp., and *Nosema* spp. Reverse transcription quantitative PCR (RT-qPCR) was performed for detection of all pathogens following previously described methods [38, 39].

Honey production and the number of colonies available to split were recorded as metrics of colony productivity. Honey production was measured by weighing supers as they were removed from colonies and is presented in total kg and kg/colony. Some splits were made directly, but the potential for splits was much higher than the actual number made because of logistical constraints of the experimental design. In order to better quantify split potential, a metric for splittable colonies was developed. A splittable colony is any colony that survived winter and had >10 frames of bees in May of the following year.

Colony mortality was assessed for three time periods per year: summer (April 1^st^–October 31^st^), winter (November 1^st^–March 31^st^), and annual (April 1st–March 31^st^). Dead colonies included colonies with zero or less than 1 frame of bees remaining, or colonies that were perpetually queenless.

### Analyses

All statistical tests were performed in R (version 3.3.3). All graphs present Average apiary data in orange and BMP apiary data in blue. All summary statistics are reported as means ± SEM unless otherwise noted. Time-series data (*i*.*e*., those collected at multiple sampling months for *Varroa*, *Nosema*, viruses, frames of bees, and brood pattern) were analyzed with mixed effects models to account for the pseudo-replication in the data. This study did not aim to describe how dependent variables changed over time or across locations, rather to describe whether management had an effect on those changes. Thus location, sampling month, and year were included as random effects in all models. Location, sampling month, and year were also included as fixed effects to test for interactions with management.

Binomial response variables (*e*.*g*., queen events, colony mortality, splits) were fitted to general binomial mixed effect models with sampling month, year, and location as random effects. When comparing variables at a single time point (*e*.*g*., at the start of the experiment) general linear models were used. Analyses of deviance were used to compare goodness of fit in a stepwise selection procedure to remove non-significant terms. A relative risk analysis was performed to assess the change in risk of annual colony mortality under a BMP regime using the following equation, and 95% confidence intervals were calculated based on approximation (R function “riskratio”, package “fmsb”):

$$RR=(\frac{{BMP}_{dead}}{{BMP}_{dead}+{BMP}_{alive}})/(\frac{{Average}_{dead}}{{Average}_{dead}+{Average}_{alive}})$$

Virus data were analyzed by prevalence (% infected) and load (copy numbers). Prevalence was analyzed with binomial mixed effects models with season, year, and location as random effects. An analysis of deviance was used to eliminate non-significant fixed effects in a stepwise fashion. Viral copy data is not suited to general linear modeling because it is highly skewed (contains a high proportion of zeros) and has large variance. Viral copy data was log-transformed to better follow a normal distribution, but the high proportion of zeros in the data still prevented general linear modelling. Rows containing zeros were then removed for each virus, and log copy numbers were analyzed for significant differences with mixed effects models. Year and location were included as random effects. An analysis of deviance was used to compare linear models to null models to generate p-values for the effect of management group. Where significant differences in viral prevalence or copy number were detected, associations with other variables including mortality, months exceeding 3.0 mites/100 bees, average yearly *Varroa* load were checked with separate mixed effects models.

## Results

### Colony strength (frames of bees, brood pattern, and queen status)

Over the 3 years, 2,244 colony strength inspections were performed. Colony health metrics were similar between management groups. The 3-year mean colony size in BMP apiaries was 11.48 ± 0.19 and in Average apiaries 11.23 ± 0.20 frames of bees. Both groups peaked in colony size in July and were smallest in October. Although frames of bees varied among years (*F*_2,1982_ = 29.0, *p* < 0.01), months (*F*_5,1982_ = 2.97, *p* = 0.02) and locations (*F*_6,1982_ = 39.6, *p* < 0.001), there was no difference between management groups (S1 Fig; *F*_1,1982_ = 0.64, *p* = 0.41).

Brood pattern was also similar between management groups. The 3-year mean brood pattern rating in BMP colonies was 3.29 ± 0.03, and Average colonies 3.26 ± 0.04. In both groups, brood pattern was lowest in fall when brood production slowed and less capped brood was present. Brood pattern varied among years (*F*_2,1892_ = 0.27, *p* < 0.05), months (*F*_5,1892_ = 10.2, *p* < 0.001), and locations (*F*_6,1892_ = 11.2, *p* < 0.001), but not between management groups (S2 Fig; *F*_1,1982_ = 0.29, *p* = 0.51).

Queen status data were subdivided into two categories: colonies that experienced a “queen event” or no “queen event.” A colony was considered to have experienced a queen event if, during colony inspection, it was found to be queenless, a drone layer, a virgin queen, or no queen or eggs were seen [40]. Colonies without queen events either had eggs present or the queen was seen. Over all 3 years, a total of 79 (39.7%) BMP colonies and a total of 83 (41.7%) Average colonies had queen events. The number of queen events differed among years (*F*_2,2003_ = 3.48, *p* = 0.05), months (*F*_5,2003_ = 2.70, *p* = 0.03), and locations (*F*_6,2003_ = 3.69, *p* < 0.01), but not between management groups (S3 Fig; *F*_1,2003_ = 0.45, *p* = 0.43). Some colonies were subject to repeated queen events, where a colony would become queenless and remain queenless for several subsequent colony inspections. There was no difference in the number of repeated queen events between management groups (*F*_1,396_ = 0.13, *p* = 0.71).

### Measures of morbidity (Varroa, Nosema, and pathogens)

#### Varroa

**Fig 2:**
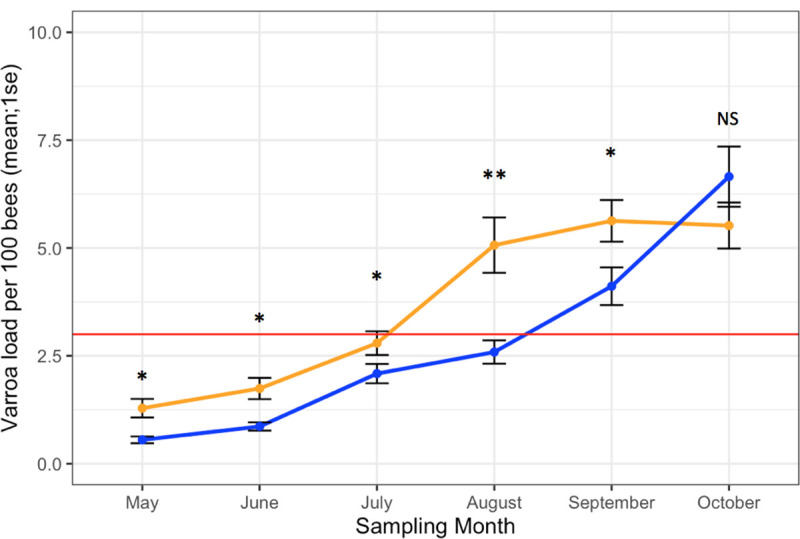
Varroa. Mean Varroa loads +/- standard error for BMP (blue) and Average (orange) apiaries over each sampling month. This graph represents all 3 years of data together. The red line represents the treatment threshold of 3.0 mites/100 bees. *p < 0.05, **p < 0.01.

BMP apiaries exhibited lower *Varroa* loads than Average apiaries across all sampling months (*F*_1,5,2017_ = 23.4, *p* < 0.001). A post hoc test showed no difference between management groups in October (*F*_1,238_ = 0.90, *p* = 0.21), indicating a convergence of *Varroa* infestation between groups after Average colonies were treated for *Varroa* in the fall. *Varroa* loads did not differ among years (*F*_2,2017_ = 0.01, *p* = 0.98). *Varroa* loads differed among sampling months, and were lowest in May and highest in October (*F*_5,2017_ = 9.25, *p* < 0.001). *Varroa* loads differed between management groups (Fig 2; *F*_1,2017_ = 10.8, *p* < 0.001), and there was a significant interaction between sampling month and management group (*F*_*1*,5,2017_ = 4.08, *p* < 0.01). *Varroa* also differed among locations (*F*_6,2017_ = 8.60, *p* < 0.001), but there was no interaction between location and management group (*F*_1_,_6,2017_ = 0.20, *p* = 0.40) with BMP apiaries exhibiting lower *Varroa* loads at each location. The 3-year average *Varroa* load in BMP apiaries was 2.67 ± 0.14 and 3.62 ± 0.18 in Average apiaries (n = 2,244).

There was no difference in Varroa load between management groups at the start of the experiment (*F*_1,238_ = 2.46, *p* = 0.12). In the second and third years, Average apiaries started the season with higher Varroa loads than BMP apiaries in May (1.24 ± 0.02 mites/100 bees compared to 0.56 ± 0.07, respectively; *F*_1,238_ = 0.93, *p* = 0.001). This inflated Varroa population persisted through each season, resulting in Average apiaries exceeding 3.0 mites/100 bees 1 sampling month prior to BMP apiaries each year. Additionally, Average apiaries spent more months above economic threshold: 1.81 ± 0.09 compared to 1.34 ± 0.08 months in BMP apiaries (*F*_1,398_ = 21.62, *p* < 0.001).

#### Pathogens

**Fig 3:**
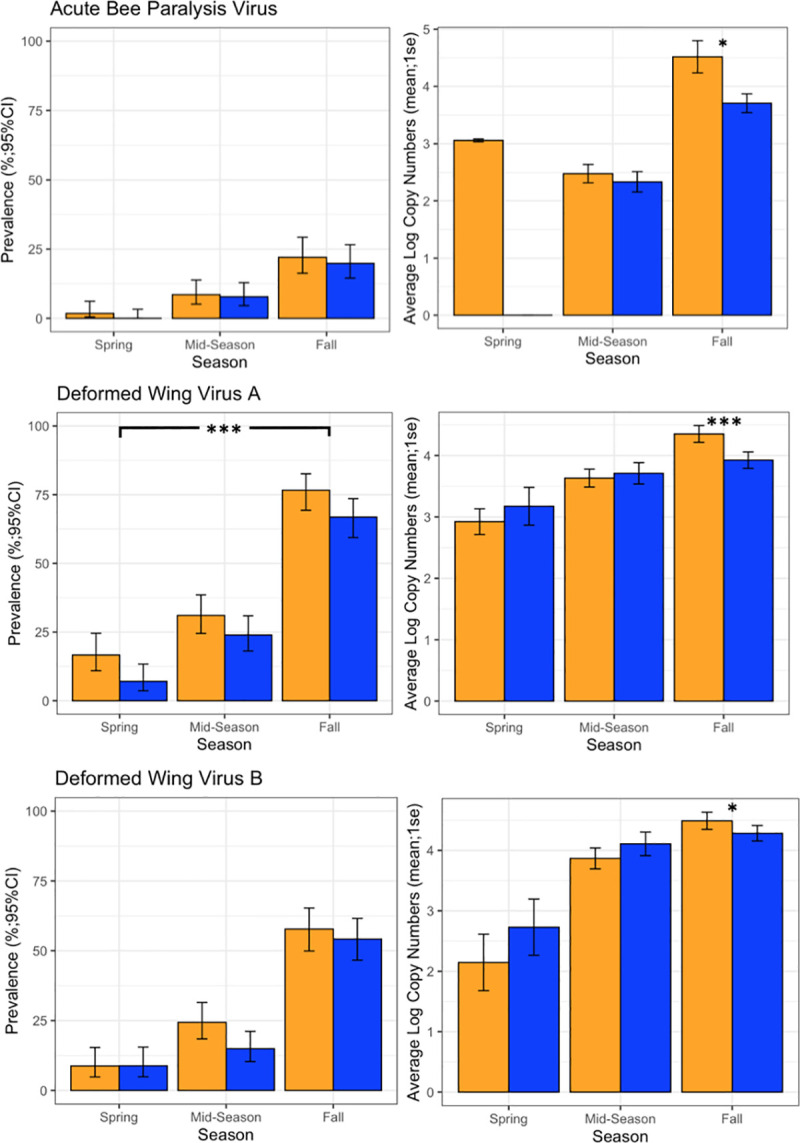
Viruses. Prevalence +/- 95% CI and Average Log Copy Numbers +/- standard error for the 3 viruses which differed between BMP (blue) and Average (orange) apiaries. These graphs represent all 3 years of data together. *p < 0.05, ***p < 0.001.

A total of 878 samples were analyzed for pathogens. Prevalence was similar between management groups, with only Deformed Wing Virus A (DWVA) being significantly lower in BMP apiaries over all seasons across all years (Fig 3; *F*_1,869_ = 3.38, *p* < 0.001). Fall load was lower in BMP apiaries for Acute Bee Paralysis Virus (ABPV) (*F*_1,258_ = 6.87, *p* = 0.01), DWVA (*F*_1,258_ = 12.89, *p* < 0.001), and DWVB (Fig 3; *F*_1,258_ = 4.30, *p* < 0.05). These metrics did not differ between BMP and Average apiaries at the start of the experiment (Prevalence: DWVA *F*_1,255_ = 1.06, *p* = 0.31; Copy Numbers: DWVA *F*_1,255_ = 2.18, *p* = 0.09; DWVB *F*_1,255_ = 2.46, *p* = 0.12; ABPV *F*_1,255_ = 0.03, *p* = 0.85), indicating that these differences developed after management practices were employed. Prevalence and loads of Black Queen Cell Virus (BQCV), Chronic Bee Paralysis Virus (CBPV), Israeli Acute Paralysis Virus (IAPV), Lake Sinai Virus (LSV), Nosema spp., and Trypanosoma spp. did not differ between management groups (S4 Fig). For the four viral metrics that significantly differed between BMP and Average apiaries (prevalence of DWVA and the fall load of ABPV, DWVA, DWVB), separate mixed effects models were performed to determine if other variables were associated with increased viral pressure. A colony’s average yearly mite load was positively associated with fall copy numbers of ABPV, DWVA, and DWVB, as well as the prevalence of DWVA (*F*_1,867_ = 21.5, *p* < 0.001; *F*_1,867_ = 18.9, *p* < 0.001; *F*_1,867_ = 23.7, *p* < 0.001; *F*_1,867_ = 25.2, *p* < 0.001, respectively). Additionally, the number of months a colony spent above 3.0 mites/100 bees was also positively associated with these same viral metrics (*p* < 0.05; *p* < 0.001; *p* < 0.01; *p* < 0.001, respectively).

#### Nosema

The 3-year average *Nosema* load in BMP apiaries across all sampling months was 0.31 ± 0.04 million spores/bee and in Average apiaries across all sampling months was 0.32 ± 0.04 million spores/bee. *Nosema* pressure in this experiment was generally low compared to other surveys [41], and averages never exceeded the commonly accepted economic threshold of 1.0 million spores/bee [42]. Average *Nosema* load in both experimental treatments followed typical *Nosema* seasonal patterns, with loads highest in spring, lowest in summer, and rising again in fall [41]. Mixed effects models showed differences among locations (*F*_6,2009_ = 7.27, *p* < 0.001) and years (*F*_2,2009_ = 0.92, *p* = 0.05), but not among sampling month (*F*_5,2009_ = 1.02, *p* = 0.17) or management groups (S5 Fig; *F*_1,2009_ = 0.03, *p* = 0.86).

### Colony outcomes (mortality, honey production, and split production)

#### Mortality

Total summer mortality for all years in BMP apiaries was 15.2% (95% CI 10.8–20.8%) and 20.6% (95% CI 15.6–26.6%) in Average apiaries. Summer mortality was highest in both groups in 2016. Binomial mixed effects models found differences among years (*F*_2,388_ = 4.77, *p* < 0.05) and locations (*F*_6,388_ = 4.42, *p* < 0.01) but no effect of management group on summer loss (*F*_1,388_ = 1.35, *p* = 0.13).

Total winter mortality for all years in BMP apiaries was 30.8% (95% CI 24.8–37.6%) and 45.2% (95% CI 38.5–52.2%) in Average apiaries. Binomial mixed effects models found differences between management groups across all years (*F*_1,388_ = 3.70, *p* < 0.01). Winter loss in Average apiaries increased each year, while in BMP apiaries winter loss decreased each year. Post hoc tests of individual years found the main reduction in winter loss in BMP apiaries occurred in 2018 (*F*_1,123_ = 7.04, *p* = 0.001). There was no interaction between location and management group (*F*_6, 388_ = 1.27, *p* = 0.09), indicating that the effects of management were similar in all locations.

**Fig 4:**
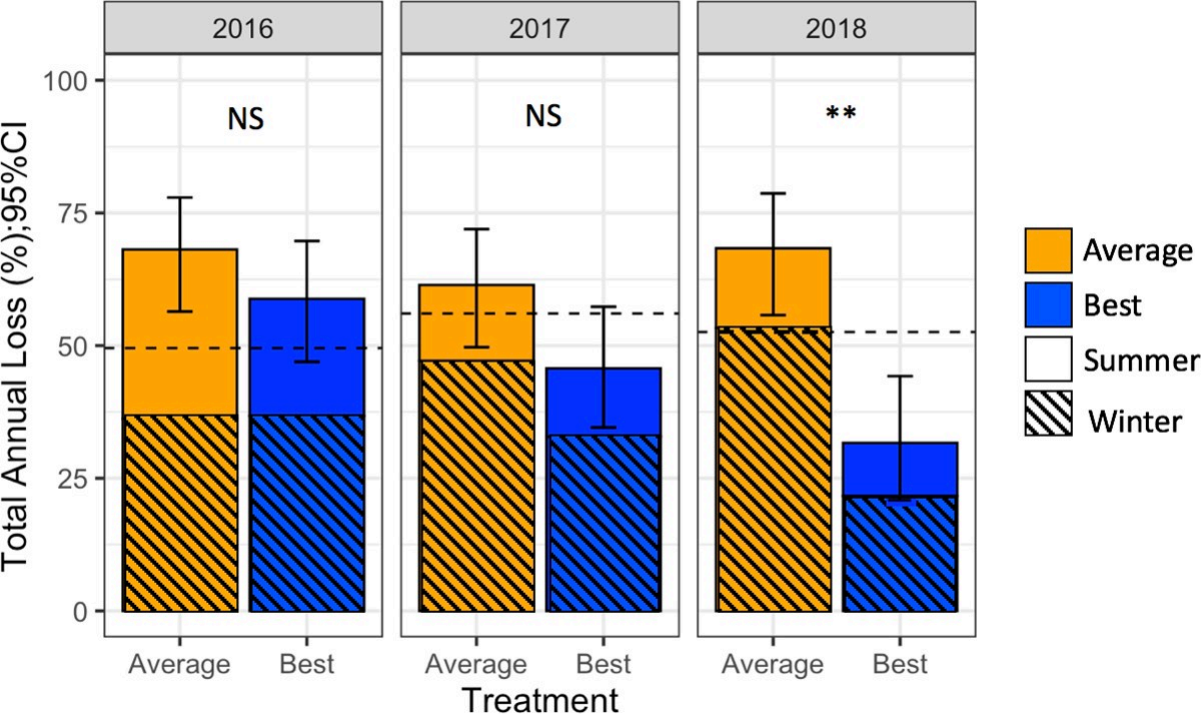
Colony mortality. Total annual loss +/- 95% CI in each BMP (blue) and Average (orange) apiaries over the 3-year experiment. Summer loss is represented by solid colors, and winter loss by striped colors. Dashed horizontal lines represent the national total winter loss for backyard beekeepers each year. **p < 0.01.

Total annual mortality for all years in BMP apiaries was 46.0% (95% CI 39.2–53.0%) and 65.8% (95% CI 59.9–72.1%) in Average apiaries. Binomial mixed effects models found no differences among locations (*F*_6,388_ = 1.03, *p* = 0.39) but did find an effect of management across all years (*F*_1,388_ = 15. 8, *p* < 0.001), with annual loss in BMP apiaries decreasing each year. A post hoc analysis of individual years found BMP apiaries lost fewer colonies in 2018 (Fig 4; *F*_1,123_ = 10.94, *p* < 0.01). A relative-risk (RR) analysis of mortality showed that using this set of best management practices reduced the risk of colony mortality by 30% (RR = 0.70, 95% CI 0.58–0.84, *p* < 0.001).

Separate binomial mixed effects models were used to check for regional differences in the effect of management on mortality. Considering separate regions is different than considering separate locations because Maryland represents one region but three locations. Regional analyses were only performed for winter and annual loss, as management had no effect on summer loss across all regions (*F*_1,388_ = 1.10, *p* = 0.21). Region did not change the effect of management on winter (*F*_4,388_ = 1.86, *p* = 0.18), or annual loss (*F*_4,388_ = 1.36, *p* = 0.24).

In Minnesota and Oregon, the number of colonies lost in BMP and Average apiaries across years was similar (S6 Fig), suggesting these management practices may not be as effective in northern climates. In Minnesota, a separate set of BMPs was tested in 2018; thus colonies from that area were not included in the 2018 analysis. Details on the Minnesota best practices and results will be published separately.

Associations between colony mortality and risk factors that differed between management groups were also assessed. A colony’s average yearly mite load was positively associated with colony mortality (*p* < 0.001). Additionally, the number of months a colony was above 3.0 mites/100 bees was positively associated with mortality (*p* < 0.001). Finally, prevalence of DWVA was positively associated with mortality (*p* < 0.05).

#### Honey production

In total, 3,699 kg of honey were harvested. Average apiaries produced a total of 1,541 kg, and BMP apiaries produced a total of 2,158 kg. No honey was harvested in 2016 as colonies had to invest significant energy in wax production in their first year (all colonies were started on foundation). The average honey produced per colony was 21.8 ± 4.6 kg and 27.2 ± 7.4 kg in Average and BMP colonies, respectively. Linear mixed effects models showed no differences between management group in the total honey produced, (*F*_1,16_ = 1.96, *p* = 0.23) mean honey produced per colony (*F*_1,16_ = 0.02, *p* = 0.85) or the proportion of colonies harvested from (*F*_1,16_ = 1.00, *p* = 0.22). BMP apiaries did produce 617 kg more honey than Average apiaries. There was a small number of BMP colonies that produced far above average honey in 2018, making the total kg produced much higher, but not significantly affecting the average produced per colony.

#### Split production

**Fig 5:**
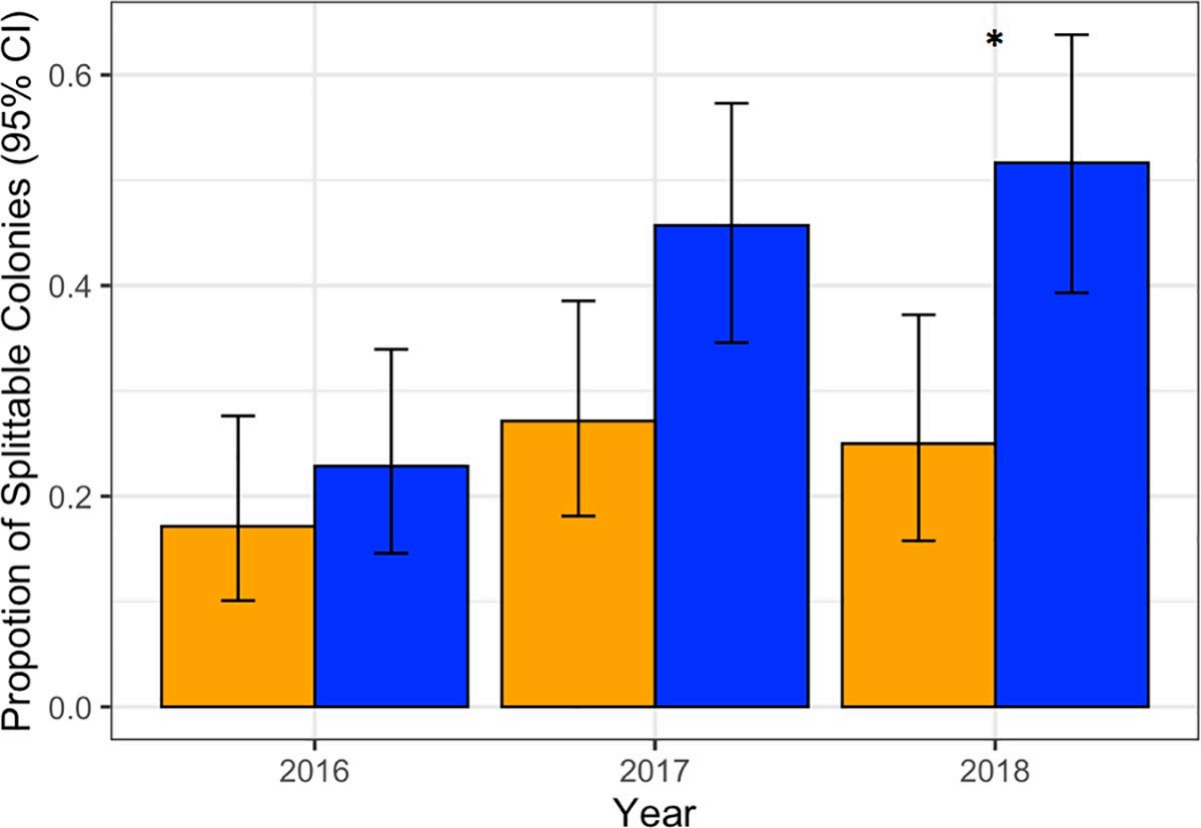
Split production. Proportion +/- 95% CI of colonies that survived winter and were splittable the following spring in BMP (blue) and Average (orange) apiaries. *p < 0.05.

Across all 3 years, BMP apiaries produced 79 splittable colonies and Average apiaries produced 46. A generalized binomial model found best apiaries produced more splittable colonies (*F*_1,388_ = 8.14, *p* < 0.01). There was an effect of year (*F*_2,388_ = 6.61, *p* < 0.05) and separate analyses conducted on each year showed that this trend increased over time. Best apiaries produce numerically more splits each year, finally producing significantly more in 2018 (Fig 5; *F*_1,123_ = 4.43, *p* < 0.05).

## Discussion

It was hypothesized that BMP apiaries would outperform Average apiaries in colony strength metrics, productivity, and survival. There were no differences between BMP and Average apiaries in colony size, brood pattern, queen status, or *Nosema* load. However, BMP apiaries did experience reduced *Varroa* loads, reaching the threshold of 3.0 mites/100 bees one month later than Average apiaries and spending fewer months above threshold overall. BMP apiaries also exhibited reduced infection levels of ABPV, DWVA, and DWVB in the fall. BMP apiaries produced more splits and experienced lower mortality than Average apiaries.

It was proposed that BMP colonies would reach larger population sizes and exhibit better queen health and productivity. BMP colonies were started from nucleus colonies or splits, which in theory should reach larger population sizes my mid-season because of greater establishment at installation. Further, it was expected that elevated fall *Varroa* loads in Average apiaries would reduce adult bee population. It seems that adult bee population during sampling months was not effected by *Varroa* load. Rather, adult bee populations may have dwindled over winter as *Varroa* loads were left unchecked in late fall, contributing to the elevated rates of colony mortality in the Average group. The similarity in colony size between management groups was unexpected but supports the idea that colony size may not represent colony health or productivity, and that other colony health metrics such as *Varroa* load and/or viral load may be better predictors of colony survival [19, 43].

The frequency of queen events between management groups was almost identical, indicating that these management practices did not affect queen issues. Brood pattern, thought to be an indicator of queen productivity, was also similar between management groups. It is surprising that Average colonies did not exhibit diminished brood patterns as a result of their elevated *Varroa* and viral loads, as these stressors often result in brood not surviving to emergence [44, 45]. However, recent work indicates brood pattern may be a result of some unknown feature of a colony’s environment as opposed to queen quality or *Varroa* or viral loads [38].

Regardless of the similarities in colony strength metrics, *Varroa* loads were significantly lower in BMP apiaries throughout the season. However, in October, mean *Varroa* population appeared to become similar between groups. One potential cause of this occurrence is horizontal transmission of mites among colonies. Horizontal transmission could have occurred if healthy colonies from BMP apiaries were robbing out weaker colonies in nearby apiaries [46]. It is known that drifting of mites and bees across colonies increases in the fall, concurrent with an increase in *Varroa* population [47]. This phenomenon may also help explain why, on occasion after miticide application, BMP apiaries reached *Varroa* loads above the economic threshold of 3.0 mites/100 bees the following month. It is unlikely that the cause of these high post-treatment mite loads is ineffective products. All experimenters used products with well demonstrated rates of mite mortality and no documented resistance. Consequently, miticides may have been effective immediately after application but the intense mite pressures within the adjacent landscape caused rapid re-infestations before the next sampling event. These re-infestations may have inflated *Varroa* measurements, so the fact that significant differences were observed in spite of this shows the effect of management is quite robust. Further, this finding emphasizes the importance of monitoring for mites as often as possible, especially after implementing control measures to ensure management effectiveness.

Despite comparable mean fall *Varroa* loads, BMP apiaries exhibited reduced winter mortality compared to Average apiaries. While post hoc tests revealed the biggest reduction in winter loss occurred in 2018, there was a significant main effect of management across all years. BMP apiaries experienced reductions in winter and annual loss in each year of the study, while Average apiaries experienced increases in winter loss and no change in annual loss. This indicates that if beekeepers adopted BMPs, they are likely to experience reduced winter losses, but these differences may not be observable until the third year.

The reduction in colony mortality may be because BMP apiaries exceeding 3.0 mites/100 bees in October would receive critical pre-winter *Varroa* management in November or December, which likely reduced mite loads below damaging thresholds. However, weather conditions did not permit sampling for *Varroa* late in the season to confirm this supposition. Still, the Average beekeeping practice of applying a single *Varroa* treatment in late summer is insufficient to adequately control mite populations in overwintering colonies.

Another consequence of insufficient *Varroa* control was demonstrated in the viral results. Prevalence of most pathogens was similar between management groups; only DWVA was less prevalent in BMP apiaries. However, the intensity of the *Varroa*-vectored viruses (ABPV, DWVA, and DVWB) in the fall was higher in Average apiaries. This suggests that Average colonies were more likely to succumb to these infections than BMP apiaries. It is possible that the elevated mite populations in Average colonies were more effective at transmitting viruses at higher rates. Models of *Varroa*-virus interactions support the hypothesis that increased mite numbers lead to increased viral load in a colony [16, 48].

Furthermore, after the first year, Average apiaries began each spring with a higher *Varroa* load than BMP apiaries, suggesting that high fall infestations from the prior year persist in a colony over winter. These *Varroa* populations remained inflated throughout the season, resulting in Average apiaries exceeding 3.0 mites/100 bees one month earlier than BMP apiaries. The number of months spent above threshold and average *Varroa* load were positively associated to viral infection and mortality. Time spent above threshold is therefore a good predictor of mortality, presumably because it is also related to viral infection. The longer a colony is above threshold, the higher the risk of experiencing *Varroa*-vectored viruses and at higher levels. This relationship can likely explain much of the mortality exhibited in Average apiaries.

An example of the effect of time spent above threshold was illustrated in Minnesota in 2017. In the first year, mite levels remained below 3.0 mites/ 100 bees in both treatment groups until mid-September when all were treated, and by the following spring, 2017, 80% of colonies in both groups survived. By July 2017, many colonies were above 3.0 mites/ 100 bees, and miticide application was delayed due to long sample processing times. As a consequence, only one colony from both management groups survived winter. To test whether the results from Minnesota were affecting our conclusions, all statistical tests above were performed with Minnesota removed from the data set. None of the significant differences detected were changed by this exclusion, indicating that the results and conclusions put forth in this study are valid regardless of the outcome in Minnesota. Another set of BMPs designed specifically for Minnesota was tested in 2018, and those results will be presented separately. The present study demonstrates the strong effect of time spent above threshold suggests that there is a cumulative effect of management and its impact on colony health. While a beekeeper can conceivably control their mite load in the fall after significant mite population build up, the damage incurred from viruses is much harder to rectify. Although monitoring all colonies every month may seem an excessive amount of work, monitoring as often as possible is just as critical early in the season as it is when preparing for winter.

The cumulative effect of management can also be seen over multiple years. The amount of honey and the number of splits produced in BMP apiaries increased each year. Winter mortality in Average apiaries increased each year, while in BMP apiaries it decreased, becoming 30 percentage points lower by the third study year. One explanation for these cumulative effects may be that new BMP colonies were started from nucs or splits in 2017 and 2018. Survey results demonstrate that nucs and splits are less likely to die than packages [30]. It is also possible that the brood break resulting from splitting overwintered BMP colonies provided extra *Varroa* control by reducing initial mite populations in parent colonies, resulting in reduced *Varroa* population growth over entire seasons [24, 49]. Another important cumulative factor is likely the elevated residual mite populations left in Average colonies in the spring of 2017 and 2018. Although mite populations in overwintered Average colonies were low enough to avoid immediate colony mortality, the overwintered mite populations negatively impacted colony health for months afterward. The resulting elevated viral loads still increased colony mortality, just over a longer time period. These results indicate that the effects of management and of colony health stressors occur over longer time periods than previously documented.

Likely as a result of reduced *Varroa* and viral pressure, the BMP apiaries outperformed Average apiaries in split production, as well as winter survival, most notably by the third year of the study. While these results seem to indicate that *Varroa* is the main driver of colony loss, and thus *Varroa* control is the only important BMP, it must be noted that the other BMPs could have contributed to colony health in subtle ways. Future tests of individual BMPs are needed to parse out their effects on colony health.

Honey production did not differ between management groups in this study. While BMP apiaries may have been expected to produce more honey, it is beneficial to confirm that these BMPs do not result in decreased honey production compared to average beekeeping practice. BMP apiaries produced 33 more splittable colonies than Average apiaries. This is likely mainly due to the larger number of BMP colonies remaining alive each spring. When factoring in the average cost that a backyard beekeeper would pay to replace a dead colony, or the price at which a beekeeper could sell a nucleus colony, these splits are worth $175 each for a total of $5,775. Furthermore, BMP practices lowered the relative risk of mortality by 30%. This represents a substantial reduction in the labor and cost of replacing dead colonies each year, assuming a beekeeper would have to replace 1/3 fewer colonies.

It is important to emphasize that this set of BMPs was specifically designed for backyard beekeepers. While elements of the results can apply to commercial operations, the logistics of such aggressive monitoring and management may only be realistic in a backyard setting. Although BMPs improved colony productivity and reduced mortality in a backyard setting, after 3 years the total loss in BMP apiaries still exceeded 30%. This is still well above the level of colony loss that beekeepers report as acceptable (~20% in 2019; [30]). This study demonstrates that while management can help inhibit some colony health stressors, it cannot prevent all colony mortality. There are environmental factors that management cannot control, such as other heavily *Varroa* infested colonies nearby, landscape nutritional quality, and pesticide exposure [17, 19, 39, 50, 51]. Even with an aggressive *Varroa*-monitoring and control strategy, BMP apiaries faced significant *Varroa* pressure and frequently exceeded economic threshold, likely as a consequence of other heavily infested colonies nearby. Indeed, supplemental feeding of carbohydrates and protein was often required and protein supplements are not as nutritious as resources from flowers [52]. Pesticide exposure could have interacted with other colony health stressors to inhibit the effects of management [53–55]. While management alone cannot prevent all colony losses, the BMPs tested in this study are meant to act as additional tools for beekeepers to bolster their colony health. This study focused on aspects of colony health that beekeepers can control, in an attempt to arm them with practical methods that can be readily integrated into their current practices to further improve colony health and reduce colony mortality across the US.

## Supporting information

S1 Table GPS coordinates of study sites. (DOCX)

S1 Fig Frames of bees. Mean frames of bee s+/- standard error for BMP (blue) and Average (orange) apiaries over each sampling month. This graph represents all 3 years of data together.
(DOCX)

S2 Fig Brood pattern. Mean brood pattern +/- standard error for BMP (blue) and Average (orange) apiaries over each sampling month. This graph represents all 3 years of data together.
(DOCX)

S3 Fig Queen events. Proportion of colonies that had a queen event, and the average number of queen events colonies had once they became queenless +/- 95% CI in BMP (blue) and Average (orange) apiaries.
(DOCX)

S4 Fig Pathogens. Prevalence +/- 95% CI and average log copy numbers +/- standard error over the season (all years combined) for viruses, Trypanosome spp. and Nosema spp. that did not significantly differ between BMP (blue) and Average (orange) apiaries.
(DOCX)

S5 Fig Nosema. Mean Nosema loads +/- standard error for BMP (blue) and Average (orange) apiaries over each sampling month. This graph represents all 3 years of data together. The red line represents the recommended economic threshold of 1.0 million spores/ bee.
(DOCX)

S6 Fig Colony mortality by region. Total annual loss +/- 95% CI in average (orange) and BMP (blue) apiaries (all years combined) by region. Summer loss is represented by solid colors, and winter loss by striped colors.
(DOCX)

S1 Data (XLSX)

S2 Data (XLSX)

S3 Data (XLSX)

S4 Data (XLSX)
