# Supplementary material for: Survey-derived best management practices for backyard beekeepers improve colony health and reduce mortality
Source: PLoS One. 2021 Jan 15;16(1):e0245490. doi: 10.1371/journal.pone.0245490 (PMC7810333; doi:10.1371/journal.pone.0245490)
Supplement: S1 Table — (DOCX) [file pone.0245490.s001.docx]

**S1 Table. GPS coordinates of study sites.**

| State | GPS Latitude | GPS Longitude |
| --- | --- | --- |
| MD (1) | 38.857649 | -76.776586 |
| MD (2) | 39.243916 | -76.930073 |
| MD (3) | 39.509853 | -77.729002 |
| MN | 44.728296 | -93.099527 |
| NC | 36.186922 | -81.246011 |
| TN | 36.112804 | -84.139889 |
| OR | 44.559630 | -123.28883 |
