# Supplementary material for: Survey-derived best management practices for backyard beekeepers improve colony health and reduce mortality
Source: PLoS One. 2021 Jan 15;16(1):e0245490. doi: 10.1371/journal.pone.0245490 (PMC7810333; doi:10.1371/journal.pone.0245490)

Supporting Figure S1. Combined 3 year mean frames of bees (+/- 1 SE) in best (blue) vs. average (orange) apiaries from May to October. Best and average apiaries did not differ in mean colony size.


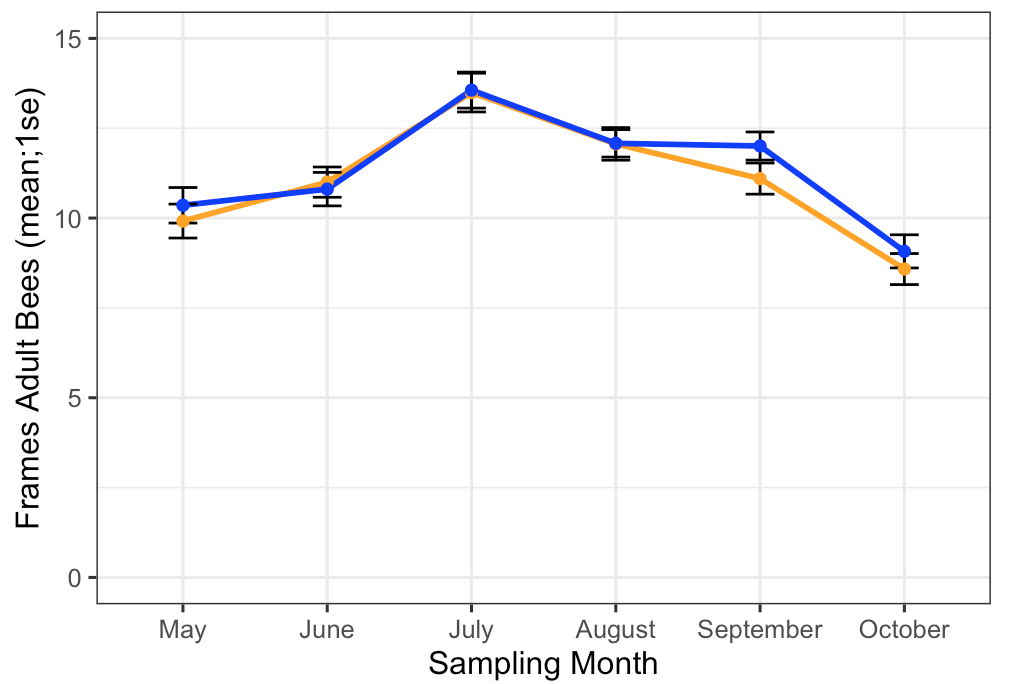

Supplement: S1 Fig — Mean frames of bee s+/- standard error for BMP (blue) and Average (orange) apiaries over each sampling month. This graph represents all 3 years of data together. (DOCX) [file pone.0245490.s002.docx]
