# Supplementary material for: Survey-derived best management practices for backyard beekeepers improve colony health and reduce mortality
Source: PLoS One. 2021 Jan 15;16(1):e0245490. doi: 10.1371/journal.pone.0245490 (PMC7810333; doi:10.1371/journal.pone.0245490)

Supporting Figure S3. Left: Mean (+/- 1 SE) number of queen events per colony over three years in best (blue) vs. average (orange) apiaries. Right: Proportion of colonies that experienced a queen event over three years in best (blue) vs. average (orange) apiaries. Best and average apiaries did not differ the number of queen events per colony or the proportion of colonies that experienced a queen event.


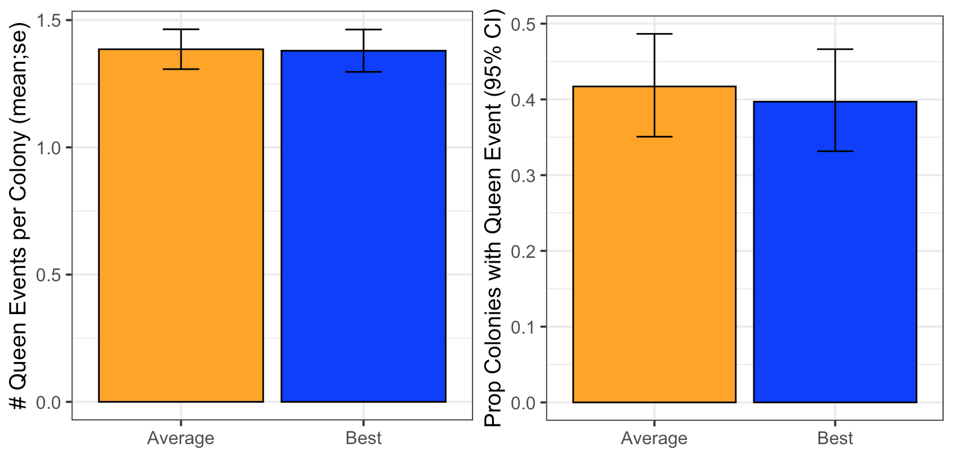

Supplement: S3 Fig — Proportion of colonies that had a queen event, and the average number of queen events colonies had once they became queenless +/- 95% CI in BMP (blue) and Average (orange) apiaries. (DOCX) [file pone.0245490.s004.docx]
