# Supplementary material for: Survey-derived best management practices for backyard beekeepers improve colony health and reduce mortality
Source: PLoS One. 2021 Jan 15;16(1):e0245490. doi: 10.1371/journal.pone.0245490 (PMC7810333; doi:10.1371/journal.pone.0245490)

Supporting Figure S1. Prevalence +/- 95% CI and average log copy numbers +/- standard error over the season (all years combined) for viruses, Trypanosome spp. and Nosema spp. that did not significantly differ between BMP (blue) and Average (orange) apiaries.

**
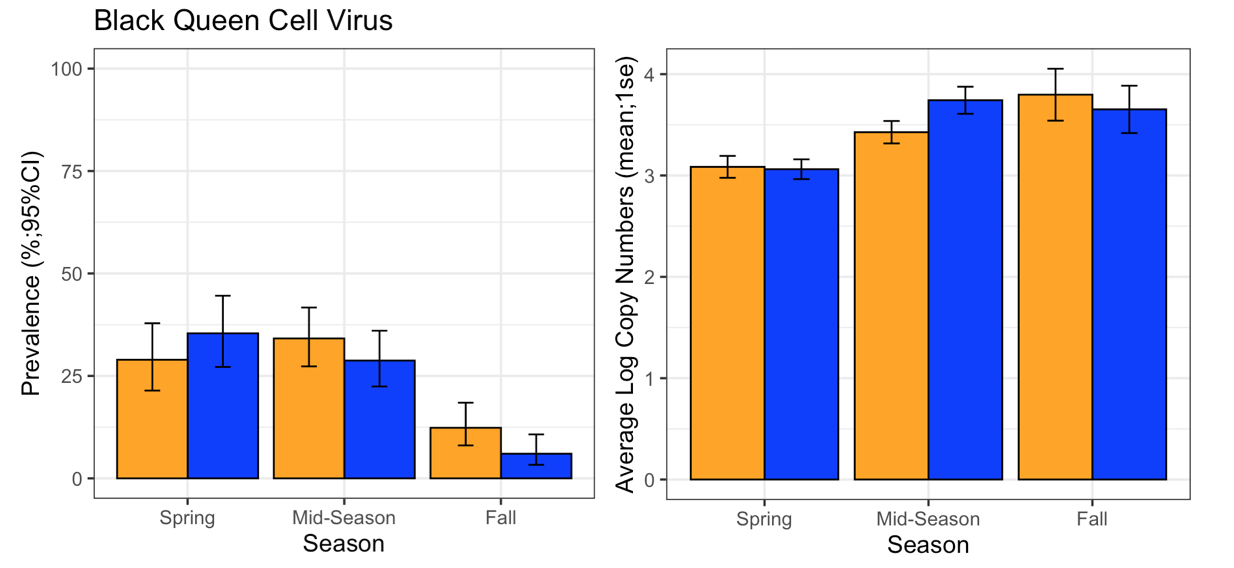
**

**
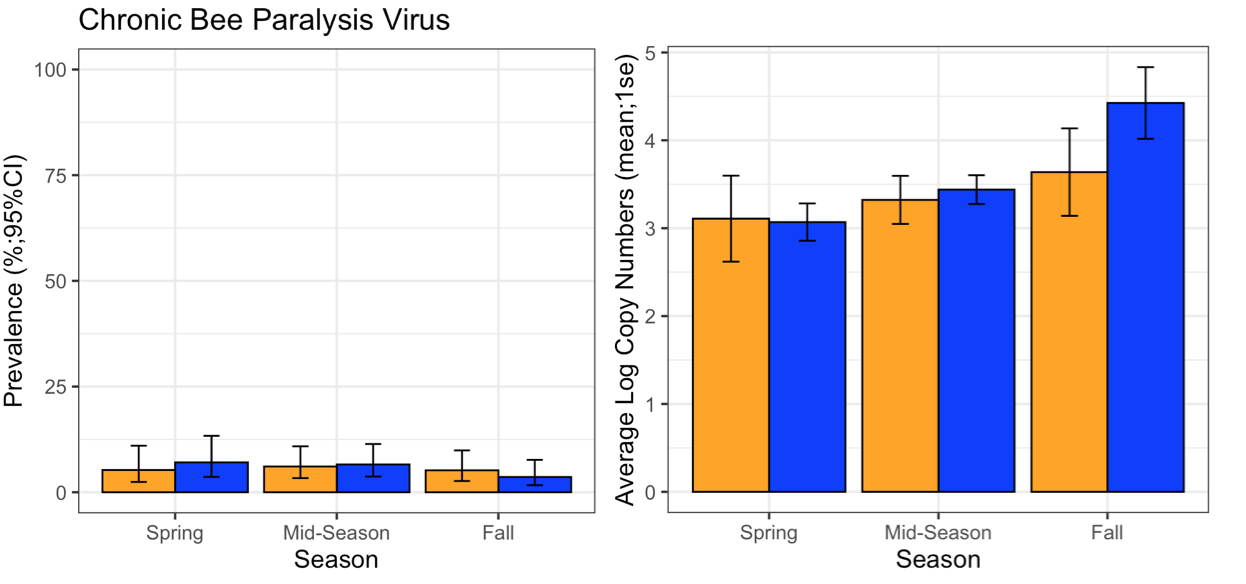
**

**
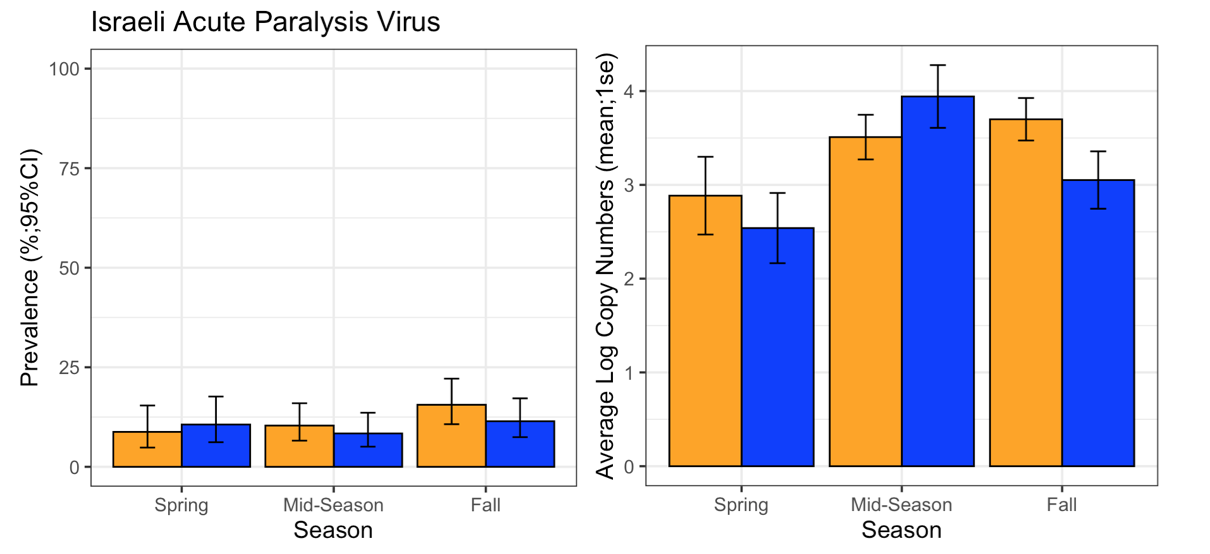
**

**
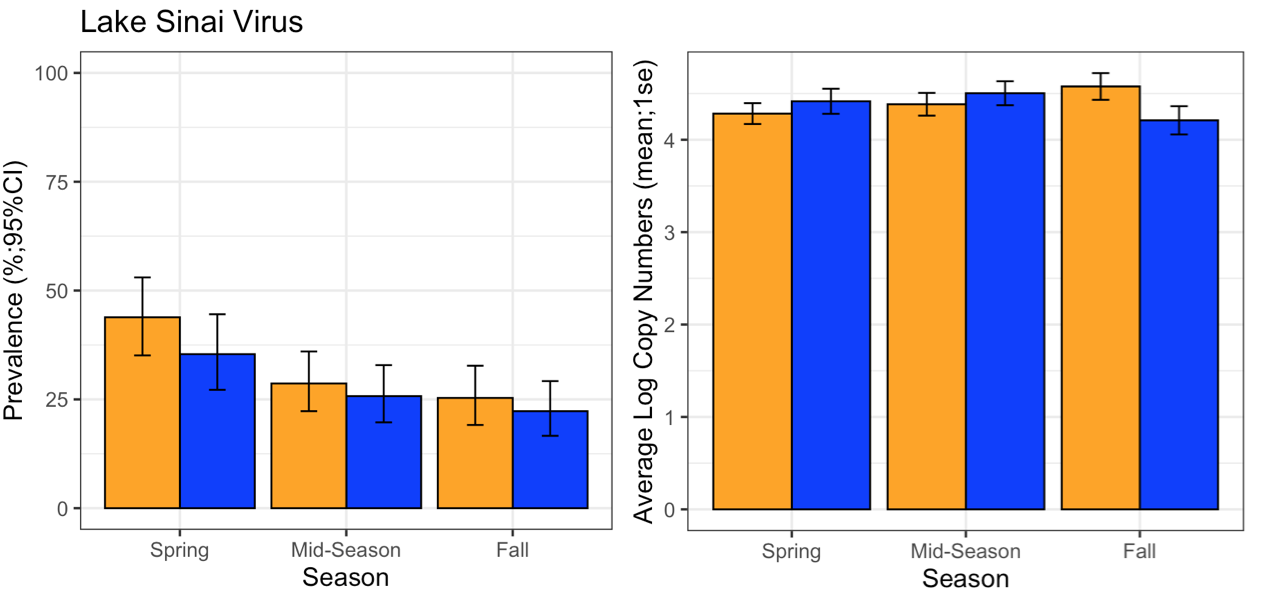
**


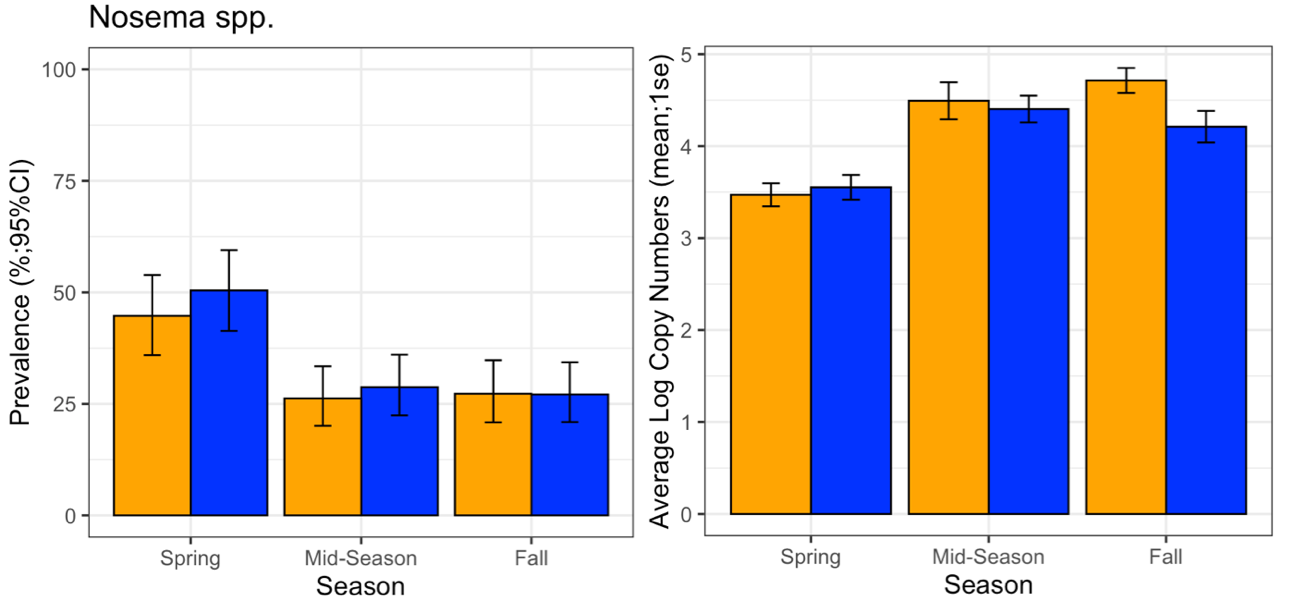


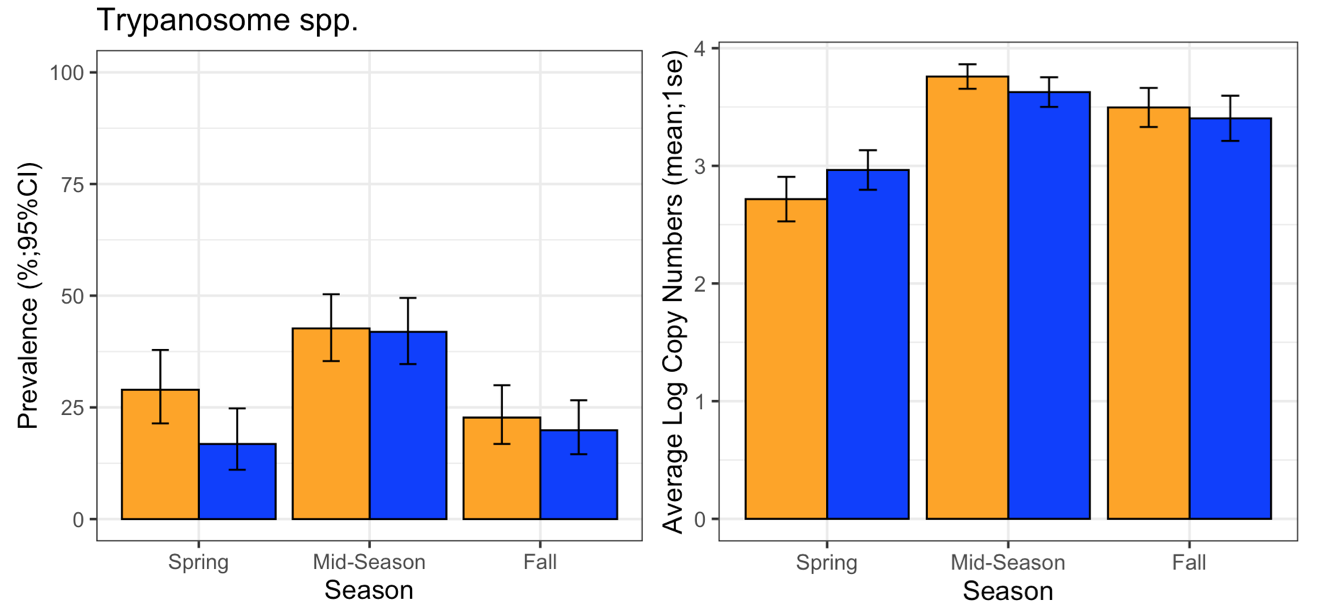

Supplement: S4 Fig — Prevalence +/- 95% CI and average log copy numbers +/- standard error over the season (all years combined) for viruses, Trypanosome spp. and Nosema spp. that did not significantly differ between BMP (blue) and Average (orange) apiaries. (DOCX) [file pone.0245490.s005.docx]
