# Supplementary material for: Survey-derived best management practices for backyard beekeepers improve colony health and reduce mortality
Source: PLoS One. 2021 Jan 15;16(1):e0245490. doi: 10.1371/journal.pone.0245490 (PMC7810333; doi:10.1371/journal.pone.0245490)

Supporting Figure S2. Total annual loss +/- 95% CI in average (orange) and BMP (blue) apiaries (all years combined) by region. Summer loss is represented by solid colors, and winter loss by striped colors


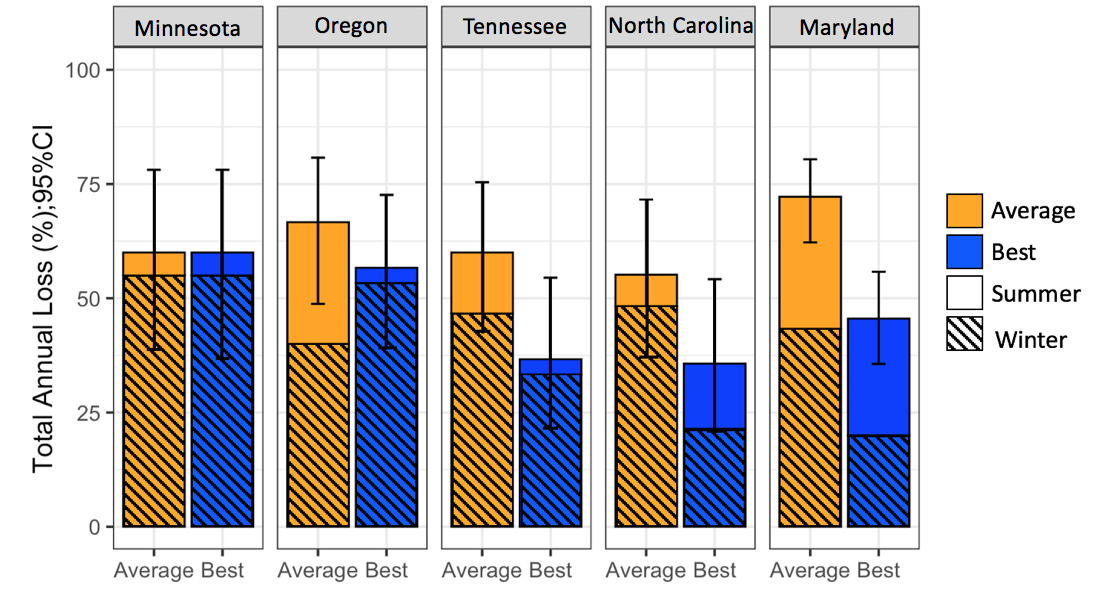

Supplement: S6 Fig — Total annual loss +/- 95% CI in average (orange) and BMP (blue) apiaries (all years combined) by region. Summer loss is represented by solid colors, and winter loss by striped colors. (DOCX) [file pone.0245490.s007.docx]
